# Jugular vein distensibility predicts fluid responsiveness in septic patients

**DOI:** 10.1186/s13054-014-0647-1

**Published:** 2014-12-05

**Authors:** Fabio Guarracino, Baldassarre Ferro, Francesco Forfori, Pietro Bertini, Luana Magliacano, Michael R Pinsky

**Affiliations:** Department of Anesthesia and Critical Care Medicine, Cardiothoracic Anesthesia and Intensive Care Medicine, Azienda Ospedaliero Universitaria Pisana, Via Paradisa, 2 56123 Pisa, Italy; Department of Anesthesia and Critical Care Medicine, Anestesia e Rianimazione Universitaria IV, Azienda Ospedaliero Universitaria Pisana, Via Paradisa, 2 56123 Pisa, Italy; Department of Critical Care Medicine, University of Pittsburgh Medical Center, Pittsburgh, PA USA

## Abstract

**Introduction:**

The purpose of the study was to verify the efficacy of using internal jugular vein (IJV) size and distensibility as a reliable index of fluid responsiveness in mechanically ventilated patients with sepsis.

**Methods:**

Hemodynamic data of mechanically ventilated patients with sepsis were collected through a radial arterial indwelling catheter connected to continuous hemodynamic monitoring system (Most Care®, Vytech Health, Padova, Italy), including cardiac index (CI) (L/min/M^2^), heart rate (beats/min), mean arterial pressure (MAP) (mmHg), central venous pressure (CVP) (mmHg) and arterial pulse pressure variation (PPV), coupled with ultrasound evaluation of IJV distensibility (%), defined as a ratio of the difference between IJV maximal antero-posterior diameter during inspiration and minimum expiratory diameter to minimum expiratory diameter x100. Patients were retrospectively divided into two groups; fluid responders (R), if CI increase of more than or equal to 15% after a 7 ml/kg crystalloid infusion, and non-responders (NR) if CI increased more than 15%. We compared differences in measured variables between R and NR groups and calculated receiver-operator-characteristic (ROC) curves of optimal IJV distensibility and PPV sensitivity and specificity to predicting R. We also calculated a combined inferior vena cava distensibility-PPV ROC curve to predict R.

**Results:**

We enrolled 50 patients, of these, 30 were R. Responders presented higher IJV distensibility and PPV before fluid challenge than NR (*P* <0.05). An IJV distensibility more than 18% prior to volume challenge had an 80% sensitivity and 85% specificity to predict R. Pairwise comparison between IJV distensibility and PPV ROC curves revealed similar ROC area under the curve results. Interestingly, combining IJV distensibility more than 9.7% and PPV more than 12% predicted fluid responsiveness with a sensitivity of 100% and specificity of 95%.

**Conclusion:**

IJV distensibility is an accurate, easily acquired non-invasive parameter of fluid responsiveness in mechanically ventilated septic patients with performance similar to PPV. The combined use of IJV distensibility with left-sided indexes of fluid responsiveness improves their predictive value.

**Electronic supplementary material:**

The online version of this article (doi:10.1186/s13054-014-0647-1) contains supplementary material, which is available to authorized users.

## Introduction

Increasing cardiac output by volume expansion is a cornerstone treatment of critically ill patients with sepsis presumed to have tissue hypoperfusion. Fluid resuscitation is performed because it is assumed that the heart is operating of the steep ascending portion of the Frank-Starling curve (preload-responsive). However, fluid resuscitation in the non-preload-responsive patient may be deleterious if it promotes *cor pulmonale*, pulmonary edema, or peripheral edema [1]. It is therefore useful to have reliable predictors of volume responsiveness.

Several studies have emphasized the reduced clinical value of static hemodynamic parameters, such as central venous pressure (CVP) and pulmonary artery occluding pressure, as compared with dynamic parameters in predicting fluid responsiveness [2,3]. Such dynamic indicators include positive-pressure ventilation-induced changes in left ventricular stroke volume and arterial pulse pressure (PP) [4]. Similarly, ultrasound evaluation of respiratory variations of both superior and inferior vena cava diameter accurately reflects volume responsiveness [5,6]. Specifically, both the superior vena cava (SVC) collapsibility index, calculated as the ratio of the difference in maximal diameter at expiration and the minimal diameter at inspiration to maximal diameter with insufflation, and the inferior vena cava (IVC) distensibility index, calculated as the difference in maximal diameter at inflation and minimal diameter at expiration predict volume responsiveness [6]. IVC imaging can be problematic in the obese and those with ascites, and SVC imaging, though more accurate requires transesophageal echocardiography, limiting its application.

Many clinical examples, from tricuspid regurgitation to heart failure, from right heart failure to both hypo- and hypervolemia illustrate that any time pressure and volume change within the intrathoracic systemic venous compartment a change also occurs in extrathoracic veins, such as in the intra-abdominal IVC or extra-thoracic internal jugular vein (IJV) [7-10]. Based on this linkage of intrathoracic venous pressure and volume to extrathoracic venous pressure we hypothesized that right heart functional status relative to its volume responsiveness should be reflected by changes in IJV pressures as assessed by IJV diameter changes.

Since IJV imaging does not require transesophageal echocardiography and is technically easier to perform than IVC visualization, we tested the hypothesis that respiratory changes in IJV diameter in mechanically ventilated patients would also predict fluid responsiveness.

## Materials and methods

After approval from the ethical committee for human biomedical of Azienda Ospedaliero Universitaria Pisana and the University of Pittsburgh, a prospective study was established elaborating hemodynamic data obtained from patients presenting sepsis, according to the definition of, and treated following the indications of the Surviving Sepsis Campaign [11]. Patients with history of any cardiac disease, evidence of jugular vein thrombosis or atrial fibrillation were excluded. Informed consent was obtained from all patients.

All enrolled patients older than 18 years were mechanically ventilated in mandatory minute ventilation (MMV) modality in supine position with the head elevated to 30°, and with ventilatory parameters adjusted to maintain Pplat <30 cmH_2_O (median 20 cmH_2_O, IQR 18 to 22), PCO_2_ <40 mmHg (respiratory rate median 16 breaths per minute, IQR 12 to 17), SaO_2_ >96%, with a tidal volume of 6 to 8 ml/kg, positive end-expiratory pressure (PEEP) of 6 cmH_2_O (median 6 cmH_2_O, IQR 5 to 7) and inspired oxygen fraction (FiO_2_) of 0.4.

We analyzed a series of measured hemodynamic variables from an indwelling radial arterial catheter in septic patients. These data included cardiac index (CI) (L/min/M^2^), heart rate (beats/minute), mean arterial pressure (MAP) (mmHg), CVP (mmHg) and pulse pressure variation (PPV) using the the Most Care® (Vytech Health, Padova, Italy) continuous hemodynamic monitoring system based on the pressure recording analytical method (PRAM) algorithm [12]. PPV is defined as the ratio of the maximum difference in PP observed over three respiratory cycles and the average of these two PPs as follows:

$$ \left(P{P}_{max}-P{P}_{min}\right)/\left(\left(P{P}_{max}+P{P}_{min}\right)/2\right). $$

A PPV >13% was presumed to identify those patients who would increase their cardiac output by >15% in response to a 500-ml colloid fluid bolus, as previously validated [4].

We simultaneously collected the hemodynamic data and an ultrasound (US) examination of the IJV by the same operator. The ultrasound examination was performed with a 12-MHz linear transducer (Esaote, Milan, Italy) and ultrasound system (MyLab™ 50 XVision). The IJV was visualized with two-dimensional echo at the level of the cricoid cartilage and recognized by compression, color Doppler and pulse wave Doppler sampling. As the patient position can influence IJV, all measurements were performed in the semi-recumbent position (head elevated 30°).

**Figure 1:**
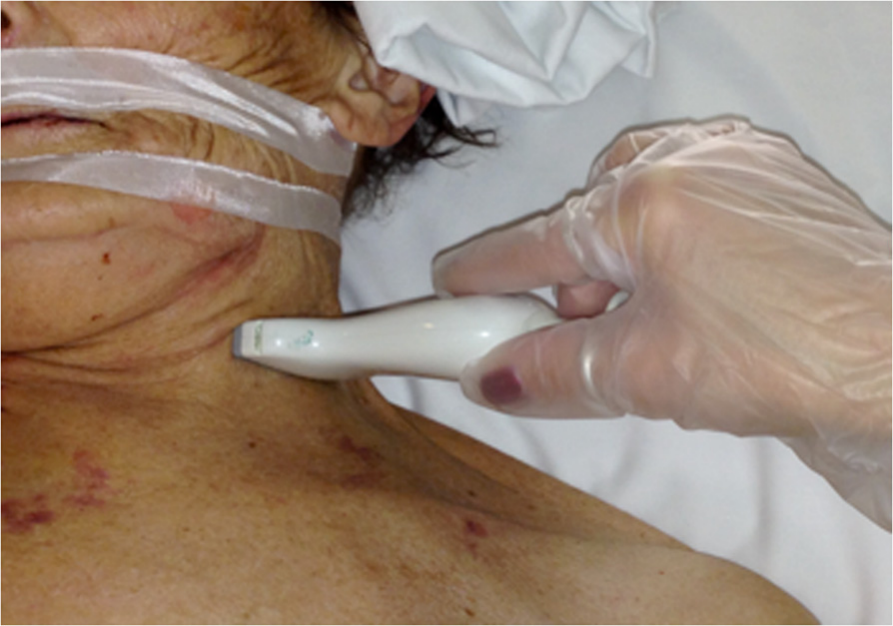
**Ultrasound probe position for internal jugular vein detection at the cricoid cartilage level.** The patient is in the supine position at 30° with head rotation of 30°.

The antero-posterior (AP) IJV diameter was measured using M-mode during a respiratory cycle. In order to avoid changes in vein diameter unrelated to respiratory variation, gentle pressure by the US probe was used to collapse the IJV in order to distinguish it from the carotid artery, then the pressure was relieved to the US probe-skin interface and attention was given to avoid influence of probe compression on IJV dimensions [13] during the US examination. Moreover, in order to avoid interference of probe-to-vein angle, the JV evaluation was performed by positioning the probe perpendicular to the skin and oriented orthogonally to the JV short-axis diameter (Figure 1). The IJV distensibility index (%) was calculated as the ratio of the difference in the maximal IJV AP diameter during inspiration and minimum IJV expiratory diameter to the minimum IJV expiratory diameter × 100.

All ultrasound measurements were performed before (T_0_) and immediately after (T_1_) 30-minute volume expansion with a 7-ml/kg crystalloid infusion. IJV distensibility was evaluated offline by an investigator blinded to the results of the fluid challenge on CI. Two consecutive measures of IJV diameters were obtained before and after fluid challenge on the first 15 patients to evaluate intraobserver variability.

Vasoactive drug infusion rates and ventilation settings were not changed during this 30-minute observation interval. Patients were defined as fluid responders (R) if an increase in CI (ΔCI) ≥15% was obtained after volume expansion, and non-responders (NR) if ΔCI was <15%.

### Statistical analysis

Data were expressed as median and IQR. The non-parametric Mann-Whitney and Wilcoxon test was used. The concordance correlation coefficient was obtained to assess intraobserver variability. Receiver operator characteristic (ROC) curves were built to obtain maximal sensitivity and specificity of PPV and IJV distensibility to predict fluid responsiveness (that is, ΔCI ≥15%). Furthermore, we analyzed the sensitivity and specificity of the combination of PPV and IJV distensibility in predicting fluid responsiveness. We classified patients in cases R or NR according to two criteria (×1, ×2) using thresholds c1 and c2. In order to compute the ROC curve, we then quantified the number of observed true positive and true negative when we classified patients to be cases if y1 < = c1 and y2 < = c2, for a variety of c1 and c2 (that is, all combinations of y1 and y2). Significance was defined by a *P*-value <0.05. Comparison between ROC curves was made using the De Long method.

## Results

From October 2012 to December 2013, we enrolled fifty septic patients (32 males and 18 females; 67, 56 to 76, median age and IQR age). None of them suffered from acute respiratory distress syndrome (ARDS) at the time of enrollment. Hemodynamic and US evaluation data are reported in Table 1. Thirty patients were R and 20 were NR. Consecutive measurements obtained in 15 patients showed substantial agreement with a concordance correlation coefficient of 0.98 (CI 0.96, 0.99) before fluid challenge and 0.98 (0.96, 0.99) after volume replacement.

**Table 1:** **Hemodynamic parameters measured in responders and non responders**

**Parameters**	**Responders**	**Non responders**	***P*** **-value**
Heart rate, time (T)0, beats/minute	6	82	0.2
	75 to 100	73 to 90	
Heart rate, T1, beats/minute	80	81	0.8
	72 to 86	71 to 88	
Heart rate change T0 to T1, beats/minute	−6	−1	0.001
	−11 to 2	−3.5 to 1.5	
Systolic pressure, T0, mmHg/ml	124	112	0.9
	110 to 135	101.5 to 142.5	
Systolic pressure, T1, mmHg/ml	137	125	0.39
	123 to 151	116.5 to 150	
Systolic pressure change T0 to T1, mmHg/ml	15	12	0.06
	11 to 20	2.5 to 16	
Cardiac index, T0, L/minute	2.2	2.45	0.18
	2.1 to 2,4	2.1 to 2.8	
Cardiac index, T1, L/minute	2.95	2.8	0.02
	2.7 to 3.3	2.27 to 3	
Cardiac index change, T0 to T1, %	36.1	5.25	<0.0001
	27.2 to 42	3.65 to 12	
Central venous pressure, T0, mmHg	9.8	10	0.15
	7.8 to 11.9	11 to 12.5	
Central venous pressure, T1, mmHg	13	13.5	0.49
	10.5 to 13.9	12 to 15	
Central venous pressure change, T0 to T1, mmHg	3	2	0.01
	2 to 3	1 to 2	
Pulse pressure variation, T0, %	22.5	12.2	<0.0001
	18 to 32	18 to 32	
Pulse pressure variation, T1, %	9.5	7.9	0.20
	7 to 16.5	5.65 to 13.5	
Pulse pressure variation change, T0 to T1, %	−14	−3.4	<0.0001
	−16 to −7	−5.2 to 2.5	
IJV distensibility T0, %	24.15	9.8	<0.0001
	20 to 29	7.6 to 13.8	
IJV distensibility, T1, %	8.9	12.2	0.07
	4.9 to 13.4	9.1 to 13.8	
IJV distensibility change T0 to T1, %	−12.5	1.1	<0.0001
	−19.4 to10.9	−1.5 to 3.1	
Norepinephrine, T0, mcg kg^−1^ min^−1^	0.1	0.1	0.5
	0.05 to 0.2	0.04 to 0.2	
Norepinephrine, T1, mcg kg^−1^ min^−1^	0.09	0.14	0.1
	0.05 to 0.2	0.0 to 0.3	

Values are expressed as median and IQR. T0 and T1 indicate before and after fluid expansion, respectively.

Basal heart rate (HR) was not different between the R and NR groups either before or after the volume challenge, though HR tended to decrease with volume challenge in R. Responders displayed an increase of systolic (*P* = 0.01) and mean (*P* = 0.05) arterial pressure, and a decrease in HR (*P* = 0.0001), with no change in diastolic arterial pressure. No significant changes in arterial pressure and no variation in HR were observed in NR group. CI increased in both groups (R, *P* = 0.0001; NR, *P* = 0.001). Despite a significant increase in CVP after fluid challenge in all patients (12, 10 to 13 mmHg to 13, IQR11, 7 to 15 mmHg, *P* = 0.0001), there was no difference in CVP between R and NR before (*P* = 0.15) or after volume expansion (*P* = 0.49). R displayed a higher variation of CVP than NR (*P* = 0.01).

**Figure 2:**
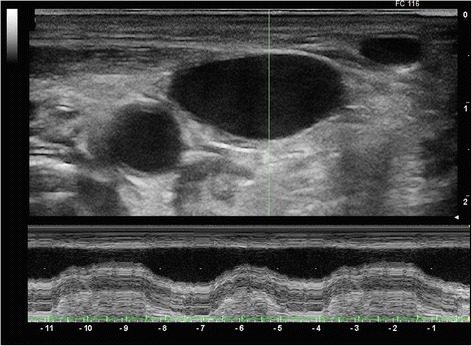
**M-mode assessment of antero-posterior diameter of the internal jugular vein (IJV) in a responsive patient under mechanical ventilation.** A high variability of IJV internal diameter is seen.

**Figure 3:**
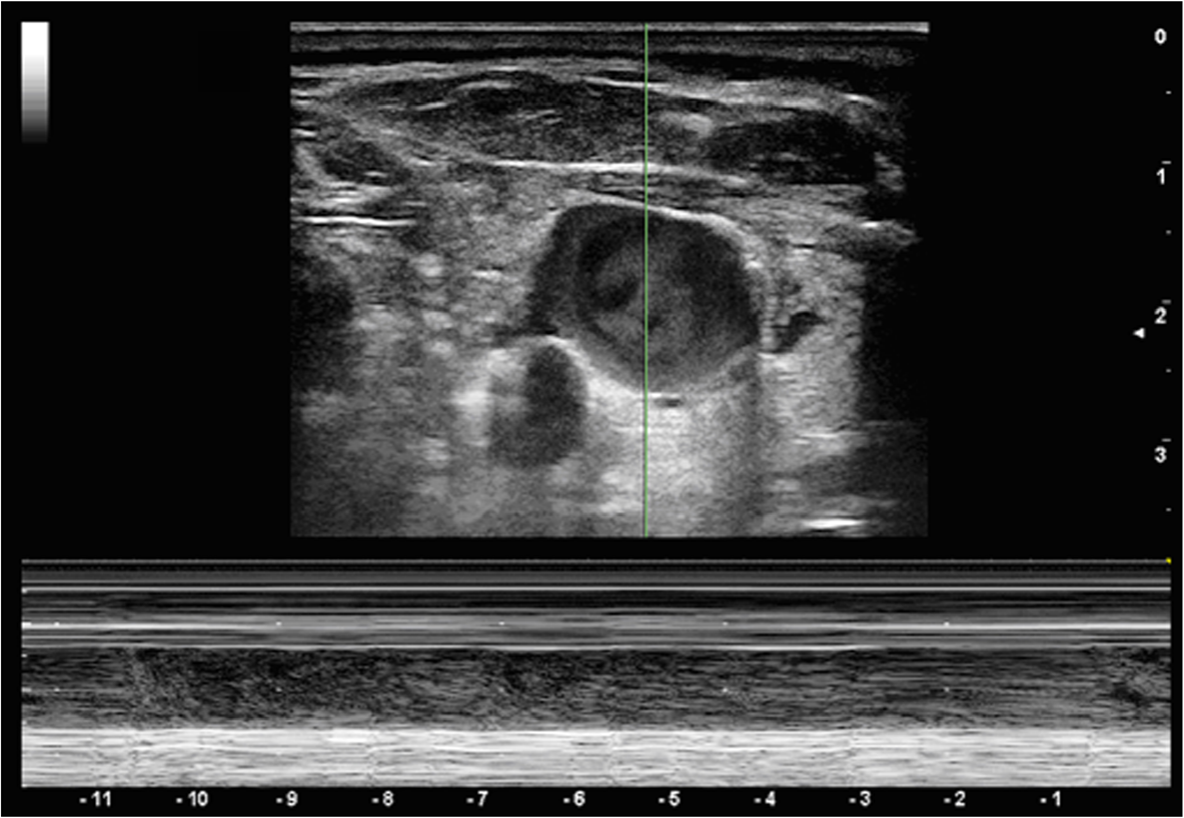
**M-mode assessment of antero-posterior diameter of the internal jugular vein (IJV) in a non-responsive patient under mechanical ventilation.** Lack of variation of IJV diameter is seen.

Responders presented higher IJV distensibility before volume expansion (Figure 2) than NR (*P* = 0.0001) (Figure 3). This difference was lost following volume challenge (*P* = 0.07). Responders had a significant reduction of IJV distensibility from baseline to post-volume expansion (*P* = 0.0001) not seen in NR (*P* = 0.26). Responders showed a higher initial PPV than NR (*P* = 0.0001). This difference was lost following volume challenge (*P* = 0.2). Both R and NR displayed a decrease in PPV with volume expansion (R and NR, *P* = 0.0001), though the decrease in PPV was greater in R (*P* = 0.0001).

**Figure 4:**
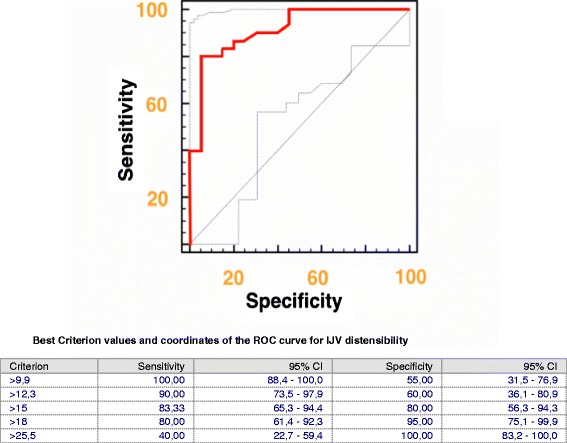
**Receiver operator characteristic (ROC) curve of internal jugular vein (IJV) distensibility before fluid administration to predict fluid responsiveness.** The gray lines represent 95% confidence bounds.

**Figure 5:**
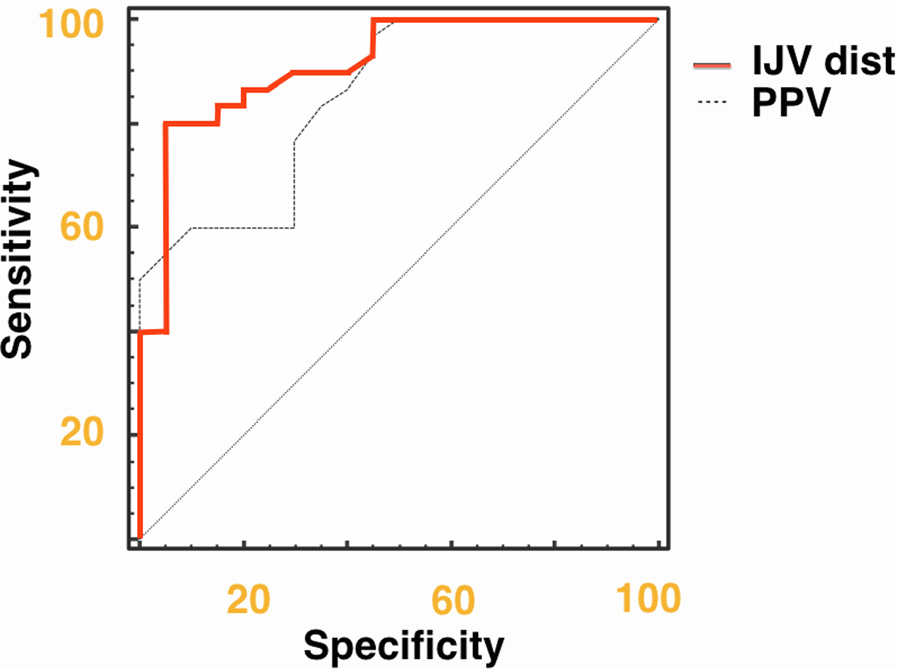
**Pairwise comparison of internal jugular vein (IJV) distensibility and pulse pressure variation (PPV) receiver operator characteristic (ROC) curves before fluid administration.**

ROC curves were constructed to establish the sensitivity and specificity of CVP, PPV and IJV distensibility in predicting fluid responsiveness. No value of CVP discriminated between R and NR with good sensitivity and specificity (area under the curve (AUC) 0,68. CI 0,45, 0,75) (see Additional file 1). A >18% IJV distensibility predicted a ΔCI ≥15% with a sensitivity of 80% and a specificity of 95%, AUC 0.915 (CI 0.801 to 0.975) (Figure 4). A value >12.5% of PPV was able to identify R with a sensitivity of 96% and a specificity of 55%, AUC 0.852 (CI 0.723 to 0.936) (see Additional file 2). We found no differences between IJV distensibility and PPV ROC curves (Figure 5). Interestingly, the combination of IJV distensibility >9.9% and PPV >12% predicted fluid responsiveness with a sensitivity of 100% and specificity of 95% (see Additional file 3).

## Discussion

The main finding of this study is that US assessment of IJV distensibility during positive-pressure ventilation can discriminate R from NR in critically ill septic patients. Furthermore, the combination of both IJV distensibility and PPV markedly increased the reliability of these predictive indices.

The use of respiratory variations of arterial pressure and aortic flow are accepted bedside parameters of fluid responsiveness and are incorporated into the display screens of many monitoring devices and used to drive resuscitation algorithms [14]. Static parameters, such as CVP, are poor predictors of fluid responsiveness as previously reported [15] and as shown in our study.

Although each dynamic measure may independently predict fluid responsiveness, combining several independently affected physiological parameters, like PPV and IJV distensibility, improves the sensitivity and specificity of these parameters to predict volume responsiveness. The improved performance of the combined measure is due, most likely, to the ability to assess both right and left sided volume responsiveness. IJV distensibility assesses venous return and right ventricular reserve while PPV assesses left ventricular response, both of which are central to controlling overall cardiovascular homeostasis.

Most of the studies evaluating the ability of functional parameters to predict the CI response to volume challenge use ROC-curve analysis to define the optimal threshold, allowing for maximum sensitivity and specificity. Recently Cannesson *et al*. used a gray-zone approach evidencing that when PPV decreases into a gray zone between 9 and 13%, uncertainty exists and clinicians should attempt to define volume responsiveness using additional measures [16]. By combining IJV distensibility with PPV, we demonstrate that such gray-zone conditions can be minimized.

Our new measure of IJV diameter change is easily seen with US with minimal training, as this approach is frequently used for US-guided central vein catheterization. We demonstrated the reliability of IJV distensibility on detecting fluid responsiveness of ventilated patients with a value of 16.4% IJV distensibility having a sensitivity of 80% and a specificity of 85% in mechanically ventilated septic patients. Similarly, PPV threshold values of 12.5% have been reported in the literature to discriminate between R and NR with similar sensitivity and specificity. Thus, both PPV and IJV distensibility can be used to assess fluid responsiveness. Perhaps more interesting, the combination of both PPV and IJV distensibility improves the sensitivity and specificity of fluid responsiveness prediction with best results for values of IJV >9.9% and PPV of >12%. These data suggest that combining right- and left-sided dynamic parameters should improve their predictive values.

Our study has several limitations. First, all subjects were on mechanical ventilation and fully adapted to the ventilator. However, IVC collapse analysis remains predictive of fluid responsiveness in spontaneously breathing subjects, suggesting that the IJV distensibility index may remain valid as well. Still, the IJV distensibility index needs to be studied in spontaneously breathing subjects. Second, we did not also measure IVC collapse or stroke-volume variation, both measures of volume responsiveness similar in quality to our IJV distensibility and PPV measures, respectively. Still, PPV should be an acceptable surrogate for stroke-volume variation in this type of comparison. Third, changes in CVP influence the IJV pressure and diameter and may decrease relative distensibility. Conditions associated with increased intra-abdominal or intrathoracic pressure may potentially increase CVP and can lead to a reduced IJV distensibility index independent of preload responsiveness. We did not study the effect of high CVP on IJV distensibility. However, this process would have similar effects on both SVC and IVC collapse parameters. Accordingly, right-sided US indices, using large-vein collapsibility to predict volume responsiveness, require careful awareness of these potential limitations. Again, these specific clinical conditions may also minimize the predictive values of PPV as well. Fourth, patients’ position can influence IJV size. The supine position leads to increased IJV diameter, which is further increased in the Trendelenburg position, whereas sitting or standing can reduce the IJV filling and IJV diameter. Therefore, we made all our measurements from a standard 30° head of the bed elevated semi-recumbent position. However, since this is the recommended position for supine ventilated patients, to minimize aspiration, this limitation needs to be understood and positioning standardized in clinical practice if IJV distensibility index is used for clinical decision-making. Fifth, we did not include patients with cardiac disease, who would particularly benefit from functional hemodynamic monitoring. However, in this initial clinical study we did not aim to evaluate the effectiveness of such methods in those patients, because of the potential confounding effect that right heart failure would impose on venous pressures. As *cor pulmonale* markedly alters other dynamic indices, often making them appear positive when not, we chose to also exclude these patients from our study. However, this patient cohort had a wide range of baseline cardiac reserve, as manifested by having only 30 of 50 patients being volume responsive. Therefore, in this initial validation study, any confounding condition, such as cardiac disease, jugular vein thrombosis or atrial fibrillation was excluded. Sixth, our only cardiovascular challenge was fluid loading. It is not clear if the use of vasoactive drugs, which may independently alter both CVP and the effective circulating blood volume, would independently affect the IJV distensibility index. This interaction and its interpretation remain to be assessed. Finally, as all measurements were performed by a single operator, inter-observer variability in IJV diameter measurement remains to be assessed in future studies.

## Conclusions

Ultrasound evaluation of IJV distensibility is a simple, easy, and readily accessible bedside measure that predicts volume responsiveness in critically ill ventilator-dependent septic patients. Importantly, the combined use of IJV distensibility with PPV increases the predictive value of these two volume-responsiveness parameters. Such right- and left-sided dynamic-measure predictor combinations need to be prospectively studied in future clinical trials.

## Key messages

- Internal jugular vein distensibility is a reliable index of fluid responsiveness in mechanically ventilated septic patients and is not inferior to pulse pressure variation
- The combined use of Internal Jugular Vein and PPV could enhance the ability to discriminate fluid responsiveness during mechanical ventilation

### Consent

Written informed consent was obtained from the patient in Figure 1 for publication of the image. A copy of the written consent is available for review by the editor-in-Chief of this journal.

## Additional files

Additional file 1:
**Area under the receiver operator characteristic (ROC) curve for central venous pressure (CVP) and pairwise comparison of ROC curves of internal jugular vein (IJV) distensibility versus CVP.**
Additional file 2:
**Best criterion values and coordinates of the receiver operator characteristic (ROC) curve for pulse pressure (PP).**
Additional file 3:
**Best values of sensitivity and specificity determined by the combination of internal jugular vein distensibility and pulse-pressure variation in predicting fluid responsiveness.**

## Abbreviations

CI: cardiac index
CVP: central venous pressure
IJV: internal jugular vein
IVC: inferior vena cava
MAP: mean arterial pressure
MMV: mandatory minute ventilation
NR: non-responders
PCO_2_: carbon dioxide partial pressure
PP: pulse pressure
PPV: pulse pressure variation
R: responders
ROC: receiver operator characteristics
SaO_2_: arterial oxygen saturation
SVC: superior vena cava
