# Supplementary material for: Jugular vein distensibility predicts fluid responsiveness in septic patients
Source: Crit Care. 2014 Dec 5;18(6):647. doi: 10.1186/s13054-014-0647-1 (PMC4301660; doi:10.1186/s13054-014-0647-1)
Supplement: Additional file 1: — Area under the receiver operator characteristic (ROC) curve for central venous pressure (CVP) and pairwise comparison of ROC curves of internal jugular vein (IJV) distensibility versus CVP. [file 13054_2014_647_MOESM1_ESM.docx]

| Area under the ROC curve (AUC) | 0,699 |
| --- | --- |
| Standard Error^a^ | 0,0741 |
| 95% Confidence interval^b^ | 0,45 to 0,75 |
| z statistic | 2,282 |
| Significance level P (Area=0.5) | 0,0225 |

^a^ DeLong et al., 1988

^b^ Binomial exact

**Criterion values and coordinates of the ROC curve** [[Hide]](javascript:hidediv('d14','d15','table1');)

| Criterion | Sensitivity | 95% CI | Specificity | 95% CI |
| --- | --- | --- | --- | --- |
| < 6 | 0,00 | 0,0 - 11,6 | 100,00 | 83,2 - 100,0 |
| <=8 | 33,33 | 17,3 - 52,8 | 100,00 | 83,2 - 100,0 |
| <=9 | 50,00 | 31,3 - 68,7 | 75,00 | 50,9 - 91,3 |
| <=10 | 73,33 | 54,1 - 87,7 | 45,00 | 23,1 - 68,5 |
| <=11 | 86,67 | 69,3 - 96,2 | 15,00 | 3,2 - 37,9 |
| <=12 | 100,00 | 88,4 - 100,0 | 0,00 | 0,0 - 16,8 |

**Pairwise comparison of ROC curves**

| IJV distensibility vs CVP | |
| --- | --- |
| Difference between areas | 0,246 |
| Standard Error^c^ | 0,0817 |
| 95% Confidence Interval | 0,0857 to 0,406 |
| z statistic | 3,008 |
| Significance level | P = 0,0026 |
| IJV distensibility vs PPV | |
| Difference between areas | 0,0633 |
| Standard Error^c^ | 0,0731 |
| 95% Confidence Interval | -0,0799 to 0,207 |
| z statistic | 0,867 |
| Significance level | P = 0,3861 |
| IJV distensibility vs CVP | |
| Difference between areas | 0,183 |
| Standard Error^c^ | 0,0944 |
| 95% Confidence Interval | -0,00261 to 0,368 |
| z statistic | 1,932 |
| Significance level | P = 0,0533 |
