# Supplementary material for: Jugular vein distensibility predicts fluid responsiveness in septic patients
Source: Crit Care. 2014 Dec 5;18(6):647. doi: 10.1186/s13054-014-0647-1 (PMC4301660; doi:10.1186/s13054-014-0647-1)
Supplement: Additional file 2: — Best criterion values and coordinates of the receiver operator characteristic (ROC) curve for pulse pressure (PP). [file 13054_2014_647_MOESM2_ESM.pdf]

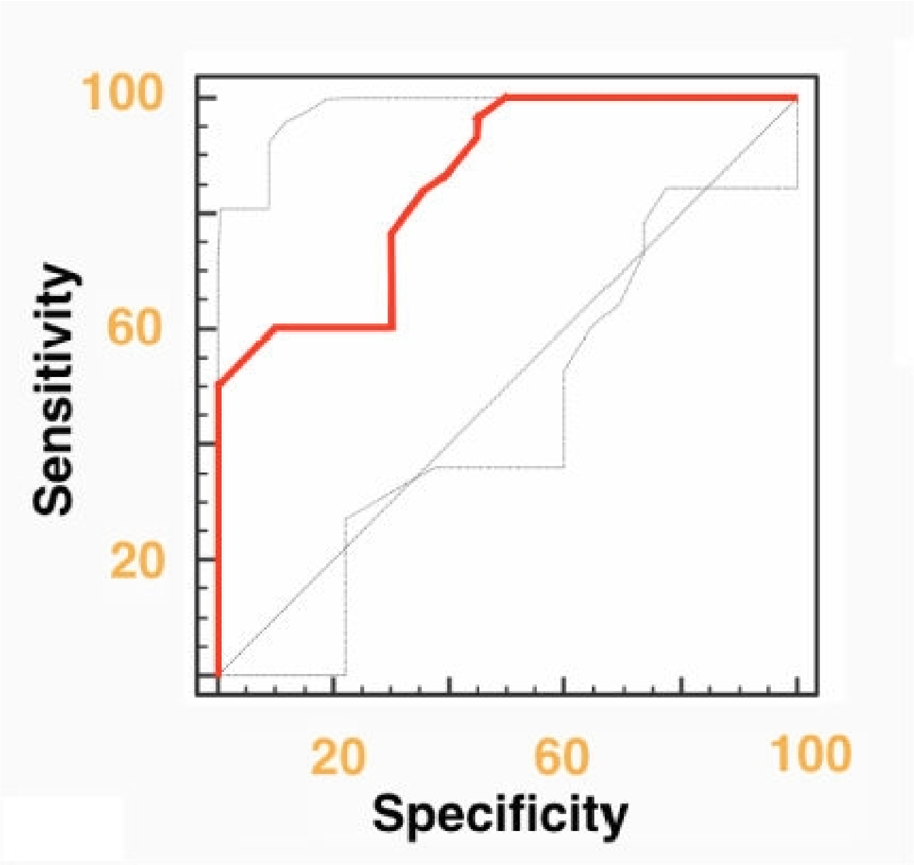

Best Criterion values and coordinates of the ROC curve for PPV

Criterion	Sensitivity	95% CI	Specificity	95% CI
>12	100,00	88,4 - 100,0	50,00	27,2 - 72,8
>12,5 *	96,67	82,8 - 99,9	55,00	31,5 - 76,9
>13,4	93,33	77,9 - 99,2	55,00	31,5 - 76,9
>14	86,67	69,3 - 96,2	60,00	36,1 - 80,9
>15	83,33	65,3 - 94,4	65,00	40,8 - 84,6
>16	76,67	57,7 - 90,1	70,00	45,7 - 88,1
>19	60,00	40,6 - 77,3	70,00	45,7 - 88,1
