# Supplementary material for: Jugular vein distensibility predicts fluid responsiveness in septic patients
Source: Crit Care. 2014 Dec 5;18(6):647. doi: 10.1186/s13054-014-0647-1 (PMC4301660; doi:10.1186/s13054-014-0647-1)
Supplement: Additional file 3: — Best values of sensitivity and specificity determined by the combination of internal jugular vein distensibility and pulse-pressure variation in predicting fluid responsiveness. [file 13054_2014_647_MOESM3_ESM.pdf]

Table 2. Best values of Sensitivity and Specificity determined by the combination of Internal Jugular vein Distensibility and Pulse Pressure Variation in predicting fluid responsiveness

Internal Jugular Vein Distensibility%	Pulse pressure Variation%	Sensitivity	1-Specificity
>16.4	>12.5	0.8	0
>16.4	>12.5	0.8	0
>16.4	>11	0.8	0
>16.4	>12	0.8	0
>13.5	>13.4	0.866667	0.05
>9.6	>12.5	0.966667	0.15
>9.6	>12.5	0.966667	0.15
>14	>12.5	0.866667	0.05
>14	>12.5	0.866667	0.05
>14	>11	0.866667	0.05
>14	>12	0.866667	0.05
>14	>12.5	0.866667	0.05
>14	>12.5	0.866667	0.05
>15.5	>13.4	0.833333	0
>9.7	>12	1	0.15
>9.9	>12	1	0.05
